# Exosomal miR-338-3p suppresses non-small-cell lung cancer cells metastasis by inhibiting CHL1 through the MAPK signaling pathway

**DOI:** 10.1038/s41419-021-04314-2

**Published:** 2021-10-30

**Authors:** Wen Tian, Xianglin Yang, He Yang, Meiwen Lv, Xinran Sun, Baosen Zhou

**Affiliations:** 1 Department of Clinical Epidemiology, First Affiliated Hospital, China Medical University, Shenyang, China; 2 Department of Epidemiology, School of Public Health, China Medical University, 110122 Shenyang, Liaoning China

**Keywords:** Non-small-cell lung cancer, Apoptosis

## Abstract

Globally, lung cancer remains one of the most prevalent malignant cancers. However, molecular mechanisms and functions involved in its pathogenesis have not been clearly elucidated. This study aimed to evaluate the specific regulatory mechanisms of exosomal miR-338-3p/CHL1/MAPK signaling pathway axis in non-small-cell lung cancer. Western blotting and qRT-PCR (reverse transcription-polymerase chain reaction) were used to determine the expression levels of CHL1 and exosomal miR-338-3p in NSCLC (non-small-cell lung cancer). The CHL1 gene was upregulated and downregulated to evaluate its functions in NSCLC progression. In vitro MTS and apoptotic assays were used to investigate the functions of CHL1 and exosomal miR-338-3p in NSCLC progression. The high-throughput sequencing was used to explore differently expressed exosomal miRNAs. The biological relationships between MAPK signaling pathway and CHL1 and exosomal miR-338-3p in NSCLC were predicted through bioinformatics analyses and verified by western blotting. Elevated CHL1 levels were observed in NSCLC tissues and cells. Upregulated CHL1 expression enhanced NSCLC cells’ progression by promoting tumor cells proliferation while suppressing their apoptosis. Conversely, the downregulation of the CHL1 gene inhibited NSCLC cells’ growth and promoted tumor cells’ apoptotic rate. Additionally, CHL1 activated the MAPK signaling pathway. Besides, we confirmed that miR-338-3p directly sponged with CHL1 to mediate tumor cells progression. Moreover, exosomal miR-338-3p serum levels in NSCLC patients were found to be low. BEAS-2B cells can transfer exosomal miR-338-3p to A549 cells and SK-MES-1 cells. In addition, elevated exosomal miR-338-3p levels significantly inhibited tumor cells proliferation and promoted their apoptosis by suppressing activation of the MAPK signaling pathway. Exosomal miR-338-3p suppresses tumor cells' metastasis by downregulating the expression of CHL1 through MAPK signaling pathway inactivation.

## Introduction

Globally, lung cancer is the leading cause of tumor-associated mortalities [1]. In 2015, an estimated 73,330 new lung cancer cases and 61,020 new lung cancer mortalities were reported in China [2]. Lung cancer is divided into two major histological types: SCLC (small-cell lung cancer) and NSCLC, with the latter accounting for 85% of all lung cancers [3]. NSCLC includes adenocarcinoma, squamous cell carcinoma, and large cell lung cancer. Lung cancer initiation and progression are closely correlated with genetic factors, which lead to abnormal mRNA and protein expressions [4]. Therefore, it is important to establish the underlying genetic mechanisms of this malignant carcinoma to inform on its treatment.

Exosomes, which range from 30 to 150 nm in diameter, have various important biomolecules, such as mRNAs, miRNAs, and lipids [5–7]. Cell-derived exosomes can change the microenvironment between cells and participate in cell metastasis [8]. Studies have reported that exosome-derived miRNAs are closely correlated with tumor development and progression [9]. In lung cancer, exosomal miR-143-3p promotes tumor cells growth by activating the PI3K/Akt signaling pathway [10]. BMSC (bone mesenchymal stem cells)-exosomal miR-193a induces NSCLC cells colony formation and metastasis by suppressing LRRC1 [11]. Elevated exosomal miR-3180-3p levels inhibit NSCLC growth and metastasis by downregulating FOXP4 [12]. In vitro and in vivo studies, exosomal miR-126 are shown to suppress ITGA6 levels, thereby promoting NSCLC development [13]. miR-338-3p has been widely reported to be involved in human cancer development and progression. In osteosarcoma, the circRNA hsa_circ_0005909/miR-338-3p/HMGA1 axis promotes tumor cells proliferation [14]; the CASC15/miR-338-3p/RAB14 axis induces tumor cells growth, migration, and invasion in vitro and in vivo [15]. In thyroid cancer, the circHIPK3/miR-338-3p/RAB23 axis is shown to activate tumor cells tumorigenesis and invasiveness in vitro [16]. Additionally, the SBF2-AS1/miR-338-3p/ADAM17 axis promotes NSCLC cells’ metastasis and proliferation [17]. However, few studies have reported on the biological role of exosomal miR-338-3p in NSCLC.

CHL1, located at 3p26, is part of the cell adhesion molecule L1 gene family that was originally reported in the nervous system [18, 19]. Emerging evidence shows that CHL1 plays a pivotal role in different tumor types [20, 21]. It has been shown to promote cells proliferation, metastasis, and migration in glioma cells [22]. In human breast cancer, CHL1 knockdown enhanced tumor cells proliferation and invasion [23]. Senchenko et al. [21] documented that the CHL1 gene is frequently upregulated in lung cancer. In our previous study, we found that CHL1 gene polymorphisms enhanced lung cancer susceptibility in northeast China [24]. Currently, the underlying molecular mechanisms of CHL1 in NSCLC still remain unclear. In this study, we aimed at evaluating the specific biological functions of the exosomal miR-338-3p/CHL1/MAPK signaling pathway axis in NSCLC.

## Results

### CHL1 is significantly upregulated in NSCLC

**Fig. 1:**
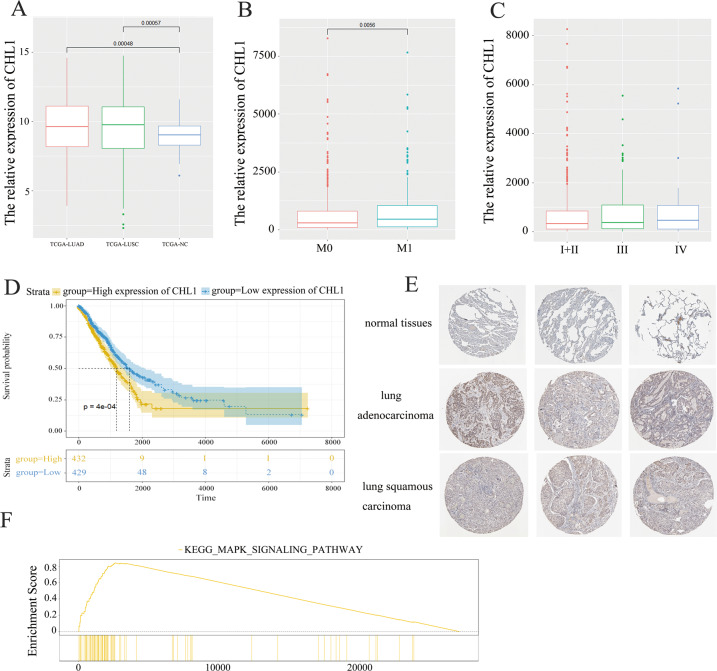
CHL1 is significantly upregulated in NSCLC. **A** The expression level of CHL1 in the TCGA-NSCLC cohort. **B** The expression level of CHL1 in patients with M0 and M1. **C** The expression level of CHL1 in patients with I + II, III, and IV. **D** The survival analysis of CHL1 in the TCGA-NSCLC cohort. **E** The immunohistochemical results of CHL1 in normal tissue, adenocarcinoma, and squamous cell carcinoma from THPA. **F** The GSEA result of CHL1 in the TCGA-NSCLC cohort.

To investigate the biological role of CHL1 in NSCLC, we acquired a CHL1 expression profile and complete clinical features from TCGA (The Cancer Genome Atlas). As Fig. 1A showed, the CHL1 gene was significantly upregulated in lung adenocarcinoma tissues (*P* < 0.001) and squamous cell carcinoma tissues (*P* < 0.001). As Table 1 listed, CHL1 abnormal expression was significantly associated with clinical stage (*P* = 0.026) and M classification (*P* = 0.001). As Fig. 1B, C showed, the expression of CHL1 was higher in advanced M classification (*P* = 0.0056) and clinical stage (*P* < 0.001). The survival analysis result showed that patients with high CHL1 expression had worse survival outcomes than those with low expression (Fig. 1D: *P* < 0.001). The results from THPA (The human protein atlas) further revealed the higher expression of CHL1 protein in adenocarcinoma and squamous cell carcinoma tissues compared to normal tissues (Fig. 1E). In addition, GSEA (gene-set enrichment analysis) result revealed that CHL1 may be enriched in the MAPK signaling pathway (Fig. 1F).

**Table 1:** The clinical characteristics of CHL1 in the TCGA-NSCLC cohort.

Characteristics	CHL1		*P* value
	Low expression	High expression	
Age			
<60	104	94	0.387
≥60	325	338	
Gender			
Male	225	202	0.095
Female	204	230	
T			
T1	109	142	0.104
T2	248	226	
T3	56	47	
T4	16	17	
N			
N0	277	286	0.787
N1	104	93	
N2	45	49	
N3	3	4	
M			
M0	337	296	**0.001**
M1	92	136	
Clinical stage			
I	203	230	**0.026**
II	146	114	
III	57	73	
IV	23	15	

Bold values indicate statistical significance.

### CHL1 promotes NSCLC cells proliferation and inhibits NSCLC cells apoptosis by inactivating MAPK signaling pathway

**Fig. 2:**
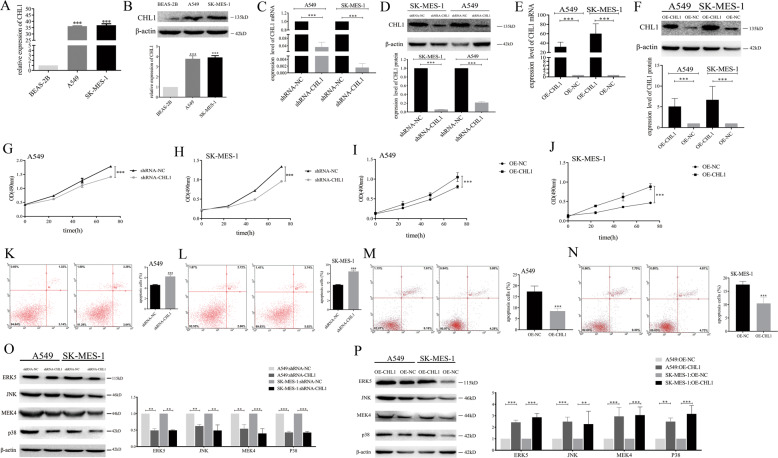
CHL1 promotes the proliferation and suppresses apoptosis of NSCLC cells by activating MAPK signaling pathway. **A** The expression level of CHL1 mRNA in BEAS-2B, A549, and SK-MES-1 cells. **B** The expression level of CHL1 protein in BEAS-2B, A549, and SK-MES-1 cells. **C** The expression level of CHL1 mRNA in A549 and SK-MES-1 cells with the treatment of shRNA-NC and shRNA-CHL1. **D** The expression level of CHL1 protein in A549 and SK-MES-1 cells with the treatment of shRNA-NC and shRNA-CHL1. **E** The expression level of CHL1 mRNA in A549 and SK-MES-1 cells with the treatment of OE-NC and OE-CHL1. **F** The expression level of CHL1 protein in A549 and SK-MES-1 cells with the treatment of OE-NC and OE-CHL1. **G** MTS assay for the growth of A549 cells with the treatment of shRNA-NC and shRNA-CHL1 at 0, 24, 48, and 72 h. **H** MTS assay for the growth of SK-MES-1 cells with the treatment of shRNA-NC and shRNA-CHL1 at 0, 24, 48, and 72 h. **I** MTS assay for the growth of A549 cells with the treatment of OE-NC and OE-CHL1 at 0, 24, 48, and 72 h. **J** MTS assay for the growth of SK-MES-1 cells with the treatment of OE-NC and OE-CHL1 at 0, 24, 48, and 72 h. **K** Apoptotic assay for A549 cells with the treatment of shRNA-NC and shRNA-CHL1. **L** Apoptotic assay for SK-MES-1 cells with the treatment of shRNA-NC and shRNA-CHL1. **M** Apoptotic assay for A549 cells with the treatment of OE-NC and OE-CHL1. **N** Apoptotic assay for SK-MES-1 cells with the treatment of OE-NC and OE-CHL1. **O** The expression levels of the MAPK signaling pathway downstream molecules with the treatment of shRNA-NC and shRNA-CHL1. **P** The expression levels of the MAPK signaling pathway downstream molecules with the treatment of OE-NC and OE-CHL1. **P < 0.01, ***P < 0.001.

First, we found that the expression levels of CHL1 mRNA (Fig. 2A: *P* < 0.001) and protein (Fig. 2B: *P* < 0.001) were higher in A549 cells and SK-MES-1 cells than those in BEAS-2B cells.

Next, to explore the function of CHL1 in tumor cells, we knocked down CHL1 expression in A549 cells and SK-MES-1 cells. The expression levels of CHL1 mRNA (Fig. 2C: *P* < 0.001) and protein (Fig. 2D: *P* < 0.001) were significantly lower in the shRNA-CHL1 groups compared with the shRNA-NC (negative control) groups. In addition, we overexpressed the expression of CHL1 and the results showed that the expression levels of CHL1 mRNA (Fig. 2E: *P* < 0.001) and protein (Fig. 2F: *P* < 0.001) were significantly higher in the OE-CHL1 groups compared with the OE-NC (overexpression negative control) groups.

In MTS assay results, the proliferation of A549 cells (Fig. 2G: *P* < 0.001) and SK-MES-1 cells (Fig. 2H: *P* < 0.001) with downregulated CHL1 level was significantly suppressed compared with the control group. Conversely, the proliferation of A549 cells (Fig. 2I: *P* < 0.001) and SK-MES-1 cells (Fig. 2J: *P* < 0.001) with upregulated CHL1 level was significantly higher compared with the control group.

Adversely, the apoptotic rates of A549 cells (Fig. 2K: *P* < 0.001) and SK-MES-1 cells (Fig. 2L: *P* < 0.001) with low CHL1 levels were significantly higher compared with the control group. Figure 2M (A549: *P* < 0.001) and 2N (SK-MES-1: *P* < 0.001) showed lower apoptotic rates of tumor cells with the treatment of OE-CHL1 compared with cells with the treatment of OE-NC.

Additionally, in order to prove the association between CHL1 and the MAPK signaling pathway, we examined the levels of the MAPK signaling pathway downstream molecules. The result showed that the expression levels of ERK5, JNK, MEK4, and p38 levels were significantly lower in cells with shRNA-CHL1 than those in cells with shRNA-NC (Fig. 2O). Additionally, the expression levels of ERK5, JNK, MEK4, and p38 levels were significantly higher in cells with the treatment of OE-CHL1 than those in cells with the treatment of OE-NC (Fig. 2P).

### miR-338-3p directly targets CHL1 by binding CHL1 3ʹ UTR

**Fig. 3:**
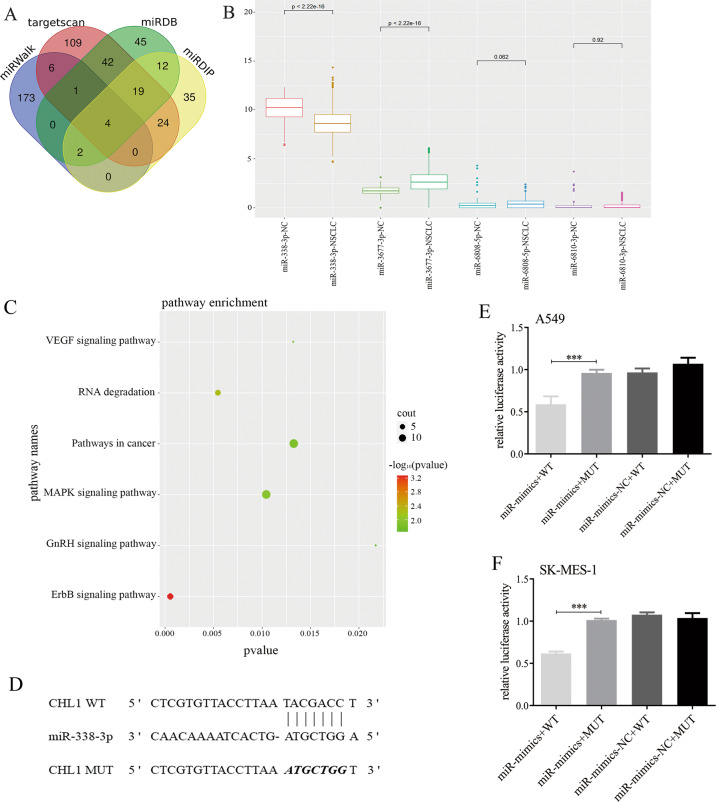
CHL1 is a direct target gene of miR-338-3p. **A** Venn diagram of target microRNAs from Targetscan, miRWalk, miRDB, and mirDIP. **B** The expression levels of target miRNAs in the TCGA-NSCLC cohort. **C** The pathway bubble diagram of miR-338-3p. **D** The binding seed sequence of miR-338-3p and CHL1 3ʹUTR. **E** The dual luciferase reporter gene assays of miR-338-3p and CHL1 in A549 cells. **F** The dual luciferase reporter gene assays of miR-338-3p and CHL1 in SK-MES-1 cells. ***P < 0.001.

To further clarify the mechanism of CHL1 in NSCLC, we predicted target miRNAs via Targetscan, miRWalk, miRDB, and mirDIP online databases. A total of four target miRNAs were collected: miR-338-3p, miR-3677-3p, miR-6808-5p, and miR-6810-3p (Fig. 3A). In the TCGA-NSCLC cohorts, the expression level of miR-338-3p in normal tissues was significantly higher than that in NSCLC tissues (Fig. 3B: *P* < 0.001).

Interestingly, as shown in Fig. 3C, miR-338-3p could be significantly enriched in the MAPK signaling pathway, suggesting that miR-338-3p might directly target CHL1 by the MAPK signaling pathway to inhibit NSCLC metastasis.

### miR-338-3p regulates tumor cells metastasis by inhibiting CHL1

**Fig. 4:**
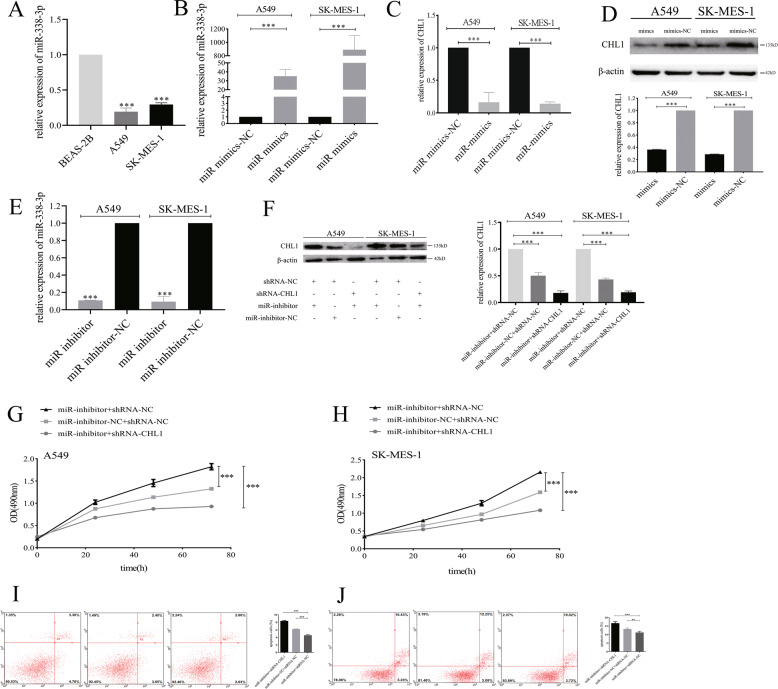
miR-338-3p regulates tumor cell growth and apoptosis by inhibiting CHL1. **A** The expression levels of miR-338-3p in BEAS-2B, A549, and SK-MES-1 cells. **B** The expression levels of miR-338-3p in A549 and SK-MES-1 cells transfected with miR-338-3p mimics and miR-338-3p mimics NC. **C** The expression levels of CHL1 mRNA in A549 and SK-MES-1 cells transfected with miR-338-3p mimics and miR-338-3p mimics NC. **D** The expression levels of CHL1 protein in A549 and SK-MES-1 cells transfected with miR-338-3p mimics and miR-338-3p mimics NC. **E** The expression levels of miR-338-3p in A549 and SK-MES-1 cells transfected with miR-338-3p inhibitor and miR-338-3p inhibitor-NC. **F** The expression levels of CHL1 protein in A549 and SK-MES-1 cells with different treatments. **G** The effect of miR-338-3p and CHL1 on A549 cells proliferation. **H** The effect of miR-338-3p and CHL1 on SK-MES-1 cells proliferation. **I** The effect of miR-338-3p and CHL1 on the apoptotic rate of A549 cells. **J** The effect of miR-338-3p and CHL1 on the apoptotic rate of SK-MES-1 cells. **P < 0.01, ***P < 0.001.

In order to elucidate the effects of miR-338-3p/ CHL1 on tumor cells, we conducted rescue experiments. A549 cells and SK-MES-1 cells were co-transfected with shRNA-CHL1 or shRNA-NC as well as miR-338-3p-inhibitor or inhibitor-NC. The result of the western blot analysis showed that the expressions of CHL1 protein were increased after downregulating miR-338-3p but that this effect was blocked by the downregulation of CHL1 (*P* < 0.001) (Fig. 4F).

MTS rescue assay revealed that the growth of cells co-transfected with miR-338-3p-inhibitor and shRNA-NC was significantly enhanced than those of cells co-transfected with miR-338-3p-inhibitor-NC and shRNA-NC. However, compared with cells co-transfected with miR-inhibitor and shRNA-NC, the growth of cells co-transfected with miR-338-3p-inhibitor and shRNA-CHL1 was significantly reduced, indicating that while the inhibition of miR-338-3p promoted the growth of tumor cells, these effects were partially reserved after CHL1 knockdown (Fig. 4G: A549: *P* < 0.001, Fig. 4H: SK-MES-1: *P* < 0.001).

Additionally, the apoptotic analyses indicated that the apoptotic rate of cells co-transfected with miR-338-3p-inhibitor and shRNA-NC was lower than those of cells co-transfected with miR-inhibitor-NC and shRNA-NC. However, compared with cells co-transfected with miR-inhibitor and shRNA-NC, the growth of cells co-transfected with miR-338-3p-inhibitor and shRNA-CHL1 was significantly induced, indicating that while the inhibition of miR-338-3p suppressed the apoptotic rates of tumor cells, these effects were partially reserved after CHL1 knockdown (Fig. 4I: A549: *P* < 0.001, Fig. 4J: SK-MES-1: *P* < 0.05).

Thus, the rescue assays proved that miR-338-3p directly targeted CHL1 to inhibit NSCLC cells growth while promoting tumor cells apoptosis.

### Exosomal miR-338-3p is significantly enriched in healthy controls serums

**Fig. 5:**
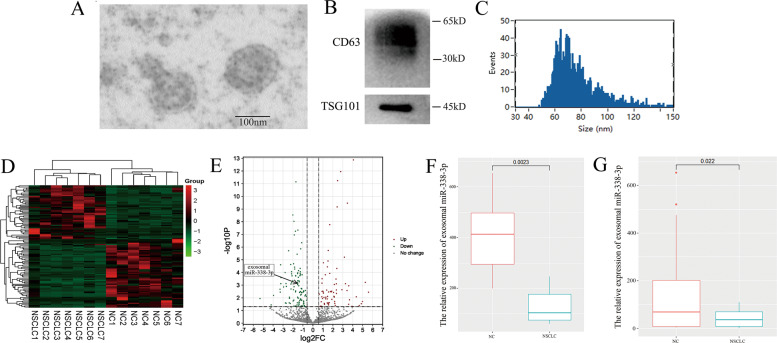
Exosomal miR-338-3p is significantly enriched in healthy controls serums. **A** The transmission electron microscope result of serum-derived exosomes. **B** The western blotting results of exosome biomarkers, CD63 and TSG101. **C** The nanoparticle tracking analysis of exosomes. **D** The heatmap of the high-throughput sequencing of serum-derived exosomes miRNAs. **E** The volcano plot of expression levels of serum-derived exosomes miRNAs. **F** The expression of exosomal miR-338-3p in 14 sample serums. **G** The expression of exosomal miR-338-3p in 66 samples of patients serums.

First, we isolated exosomes from serums and identified the cup-shaped construction and size by electron microscopy (Fig. 5A). Next, we detected the exosome biomarkers, CD63 and TSG101 (Fig. 5B). Figure 5C showed the result of nanoparticle tracking analysis, indicating that the average exosome size ranged from 50 to 120 nm. The above results verified that the isolated particles were exosomes.

We collected a total of 14 samples serums to explore the differently expressed exosomal miRNAs. A total of 111 downregulated exosomal miRNAs and 71 upregulated exosomal miRNAs were observed (Fig. 5D). Figure 5E, F showed that exosomal miR-338-3p was significantly upregulated in healthy controls serums. In order to prove the upregulation of exosomal miR-338-3p, we expanded the quantity of samples and collected another 66 samples of patients' serums. The high expression level of exosomal miR-338-3p in healthy serums was proved in Fig. 5G. As Table 2 listed, the exosomal miR-338-3p expression was significantly associated with pathological classification (*P* < 0.001), M classification (*P* = 0.018), and lymphatic metastasis (*P* = 0.030).

**Table 2:** The clinical characteristics of exosomal miR-338-3p in NSCLC patients.

Characteristics	exosomal miR-338-3p		*P* value
	High expression	Low expression	
Age			0.155
<60	16	15	
≥60	12	23	
Gender			0.833
Male	14	18	
Female	14	20	
Pathological classification			
Lung adenocarcinoma	24	14	**<0.001**
Lung squamous carcinoma	1	15	
Others	3	9	
Smoking			0.541
Yes	9	15	
No	19	23	
Drinking			
Yes	5	10	0.418
No	23	28	
T			0.566
T1+T2	16	19	
T3+T4	12	19	
M			**0.018**
M0	17	12	
M1	11	26	
Clinical stage			0.379
I+II	11	11	
III+IV	17	27	
Lymphatic metastasis			**0.030**
Yes	16	31	
No	12	7	

Bold values indicate statistical significance.

### BEAS-2B cells-derived exosomes transfer miR-338-3p to tumor cells

**Fig. 6:**
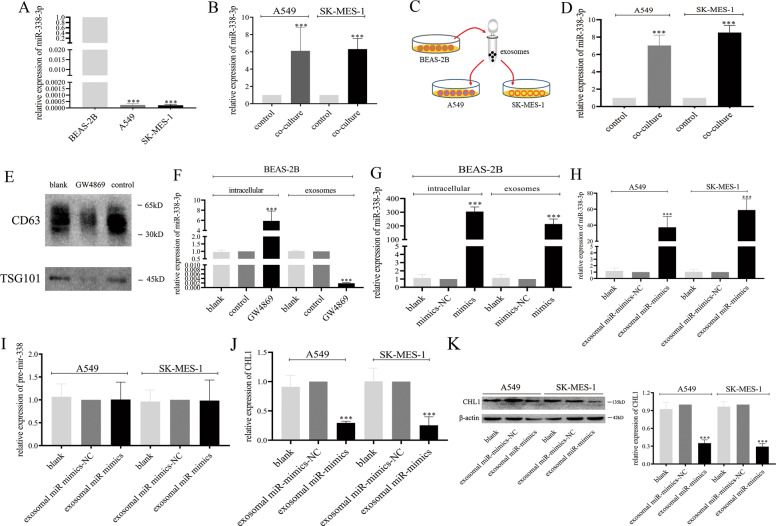
BEAS-2B cells-derived exosomes deliver miR-338-3p to A549 and SK-MES-1 cells. **A** The expression levels of miR-338-3p in BEAS-2B, A549, and SK-MES-1 cells-derived exosomes. **B** The expression levels of miR-338-3p in A549 and SK-MES-1 cells after co-culture with BEAS-2B cells. **C** Schematic of the co-culture design. **D** The expression levels of miR-338-3p in A549 and SK-MES-1 cells after co-culture with BEAS-2B cells-derived exosomes. **E** The effect of GW4869 on BEAS-2B-derived exosomes. **F** The expression levels of miR-338-3p in BEAS-2B cells and cells-derived exosomes after the treatment of GW4869. **G** The expression levels of miR-338-3p in BEAS-2B cells and cells-derived exosomes after the transfection of miR-338-3p mimics and mimics NC. **H** The expression levels of miR-338-3p in A549 and SK-MES-1 cells after co-culture with exosomes derived from BEAS-2B transfected with miR-338-3p mimics and mimics NC. **I** The expression levels of pre-mir-338 in A549 and SK-MES-1 cells after co-culture with exosomes derived from BEAS-2B transfected with miR-338-3p mimics and mimics NC. **J** The expression levels of CHL1 mRNA in A549 and SK-MES-1 cells after co-culture with exosomes derived from BEAS-2B transfected with miR-338-3p mimics and mimics NC. **K** The expression levels of CHL1 protein in A549 and SK-MES-1 cells after co-culture with exosomes derived from BEAS-2B transfected with miR-338-3p mimics and mimics NC. ***P < 0.001.

First, Fig. 6A showed that the expression levels of miR-338-3p were significantly higher in BEAS-2B cells-derived exosomes than those in A549 cells-derived exosomes (*P* < 0.001) and SK-MES-1 cells-derived exosomes (*P* < 0.001). Then we co-cultured BEAS-2B cells with A549 cells and SK-MES-1 cells and the results showed that the expression levels of exosomal miR-338-3p in A549 cells and SK-MES-1 cells in the co-culture group were significantly higher than those in the control group (Fig. 6B: *P* < 0.001). Thus, we put forward the hypothesis that BEAS-2B cells could transfer miR-338-3p to tumor cells through exosomes. As shown in Fig. 6C, we collected exosomes from BEAS-2B cells and co-cultured exosomes with A549 cells and SK-MES-1 cells. Interestingly, after co-culture, we found that the expression levels of miR-338-3p in A549 cells and SK-MES-1 cells were significantly increased (Fig. 6D: *P* < 0.001). Continually, we utilized GW4869 to suppress the secretion of exosomes from BEAS-2B cells. As shown in Fig. 6E, the expression levels of CD63 and TSG101 were significantly suppressed in the GW4869 group than those in the control group. However, no differences were observed between the control group and the blank group. Then we found that the expression level of miR-338-3p in BEAS-2B cells with treatment of GW4869 was significantly increased. Conversely, the expression level of cells-derived exosomal miR-338-3p was significantly decreased (Fig. 6F: *P* < 0.001), proving the above hypothesis that BEAS-2B cells could transfer exosomal miR-338-3p to A549 cells and SK-MES-1 cells.

Next, we transfected miR-338-3p mimics and mimics-NC to BEAS-2B cells. Higher expression levels of miR-338-3p and exosomal miR-338-3p were observed in BEAS-2B cells (Fig. 6G: *P* < 0.001). Then we collected exosomes from BEAS-2B cells transfected with miR-338-3p mimics and mimics-NC and co-cultured exosomes with A549 cells and SK-MES-1 cells. Consistently, the expression levels of miR-338-3p in A549 cells and SK-MES-1 cells were significantly increased in the co-culture groups (Fig. 6H: *P* < 0.001). Additionally, we explored the expression level of pre-mir-338 and the results showed no significant differences among tumor cells with different treatments (Fig. 6I). The above results demonstrated that A549 cells and SK-MES-1 cells could directly take up miR-338-3p through exosomes secreted from BEAS-2B cells.

Besides, Fig. 6J, K showed that the levels of CHL1 mRNA and protein were significantly inhibited in cells co-cultured with exosomes with miR-338-3p mimics than those in cells co-cultured with exosomes with miR-338-3p mimics-NC (Fig. 6J: *P* < 0.001, Fig. 6K: *P* < 0.001), further demonstrated that exosomal miR-338-3p could reduce the expression of CHL1 in NSCLC.

### Exosomal miR-338-3p regulates tumor cells proliferation and apoptosis

**Fig. 7:**
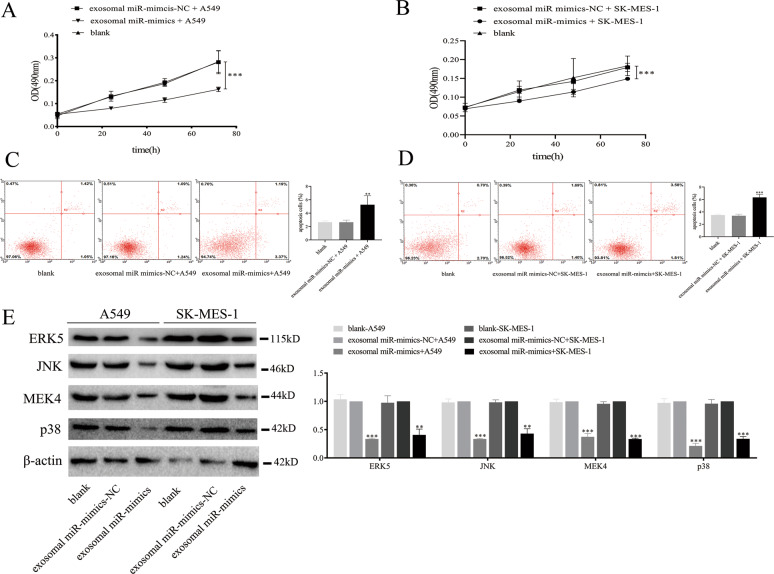
Exosomal miR-338-3p suppresses NSCLC cell proliferation and promotes NSCLC cell apoptotic rate by inactivating MAPK signaling pathway. **A** MTS assay of the growth in A549 cells with different treatments at 0, 24, 48, and 72 h. **B** MTS assay of the growth in SK-MES-1 cells with different treatments at 0, 24, 48, and 72 h. **C** Elevated exosomal miR-338-3p levels promoted the apoptotic rate of A549 cells. **D** Elevated exosomal miR-338-3p levels promoted the apoptotic rate of SK-MES-1 cells. **E** The expression levels of the MAPK signaling pathway downstream molecules with different exosomal miR-338-3p levels. **P < 0.01, ***P < 0.001.

To verify the role of exosomal miR-338-3p in NSCLC, A549 cells and SK-MES-1 cells were co-cultured with exosomes from BEAS-2B cells transfected with miR-338-3p mimics and mimics-NC. MTS results showed that the growth rates of cells with higher exosomal miR-338-3p expression were lower (Fig. 7A: A549: *P* < 0.001; Fig. 7B: SK-MES-1: *P* < 0.001). Apoptotic assay results showed that the apoptotic rates of cells with elevated exosomal miR-338-3p expression were higher than the control groups (Fig. 7C: A549: *P* < 0.001; Fig. 7D: SK-MES-1: *P* < 0.001). However, no significant differences were observed between the blank group and the NC group.

To further verify the effect of exosomal miR-338-3p on the MAPK signaling pathway, we investigated the expression levels of its downstream molecules with different treatments. As shown in Fig. 7E, the expression levels of ERK5, JNK, MEK4, and p38 levels were significantly decreased in tumor cells with higher exosomal miR-338-3p expression.

## Discussion

Pathogenesis of non-small-cell lung cancer (NSCLC) has not been clearly established. Apart from environmental factors, genes and microRNAs play important roles in cancer development and progression. Studies have reported that CHL1 promotes breast cancer [23] and esophageal squamous cell carcinoma [25] cells proliferation. In addition, studies have reported that CHL1 levels in NSCLC tissues are significantly elevated and are closely associated with T stage and metastatic lymph node status [26]. In this study, our data revealed the elevated CHL1 levels were significantly associated with survival outcomes in NSCLC. Moreover, CHL1 knockdown significantly suppressed tumor cells proliferation and promoted apoptosis and CHL1 overexpression promoted tumor cells proliferation and inhibited apoptosis, demonstrating that CHL1 acts as an oncogene in NSCLC metastasis.

Studies have reported that miR-338-3p is involved in the development and progression of human cancers. In epithelial ovarian cancer, miR-338-3p levels were found to be significantly suppressed and were closely correlated with poor prognosis [27]. In bladder cancer, upregulated miR-338-3p suppressed tumor cells proliferation, metastasis, and EMT (epithelial−mesenchymal transition) [28]. In colorectal cancer, miR-338-3p targets MACC1 to modulate tumor cells proliferation, migration, and apoptosis [29]. In prostate cancer, miR-338-3p inhibits tumor cells tumorigenicity by inactivating RAB23 [30]. In lung cancer, miR-338-3p directly binds Sox4 to silence tumor cells’ metastasis [31]. Moreover, RAB14, one of the targeted genes of miR-338-3p, acts as a cancer promoter in NSCLC [32]. In this study, we predicted target miRNAs of CHL1 through bioinformatics analyses. The rescue experiments demonstrated that the effect of suppressed miR-338-3p levels on tumor cells proliferation and apoptosis was rescued by inhibited CHL1 levels. Moreover, exosomal miR-338-3p could directly suppress CHL1 levels, indicating that exosomal miR-338-3p participates in the growth and apoptosis of NSCLC by suppressing the CHL1 level.

The MAPK signaling pathway plays a pivotal role in cells growth, differentiation, and apoptosis [33]. It has been previously reported that CHL1 downregulation activates ERK1/2 to regulate neuronal cells growth [34]. Another study documented that CHL1 regulates neuroblastoma cells’ proliferation and differentiation by inhibiting the MAPK signaling pathway [35]. In our study, we predicted that CHL1 was significantly enriched in the MAPK signaling pathway. Silenced CHL1 expression significantly inhibited the activation of the MAPK signaling pathway and upregulated CHL1 expression activated MAPK signaling pathway, proving that CHL1 promotes NSCLC cell proliferation and reduces apoptosis through activating the MAPK signaling pathway.

Previous studies have reported that exosomal miRNAs function as biomarkers in NSCLC. Low exosomal miR-382 level was significantly associated with OS of patients with NSCLC [36]. Circulating exosomal miR-96 was significantly upregulated and correlated with OS of patients, indicating that exosomal miR-96 could be considered as a novel biomarker in diagnostic and prognostic of NSCLC [37]. Exosomal miR-620 was downregulated and associated with distant metastasis and chemotherapeutic effect of patients with early-stage NSCLC [38]. In our study, exosomal miR-338-3p was downregulated in NSCLC serum samples. Additionally, we found a significant association between exosomal miR-338-3p and different clinical characteristics in NSCLC, implying that exosomal miR-338-3p functioned as a promising diagnostic and therapeutic biomarker of patients with NSCLC.

Multiple studies have demonstrated that donor cells can transfer miRNAs to recipient cells through exosomes to regulate the development and progression of NSCLC. Exosomal miR-155 and exosomal miR-196a-5p derived from tumor-associated macrophages activated NSCLC metastasis [39]. Exosomal miR-224-5p derived from tumor cells significantly promoted the growth and metastasis of NSCLC by inhibiting AR expression [40]. miR-3180-3p-enriched exosomes reduced the progression of NSCLC by suppressing FOXP4 expression [12]. In our study, BEAS-2B cells could directly transfer miR-338-3p to NSCLC cells through exosomes to regulate the growth and apoptotic rates of recipient cells.

The biological link between miR-338-3p and the MAPK signaling pathway was also reported. For osteosarcoma cells, miR-338-3p inhibits RUNX2/CDK4 expression and the MAPK signaling pathway to inactivate tumor cells proliferation and metastasis [41]. Moreover, in cervical cancer, miR-338-3p regulates tumor cells growth in MACC1 through the MAPK signaling pathway [42]. Consistently with present studies, elevated exosomal miR-338-3p levels significantly inhibited the activation of the MAPK signaling pathway.

**Fig. 8:**
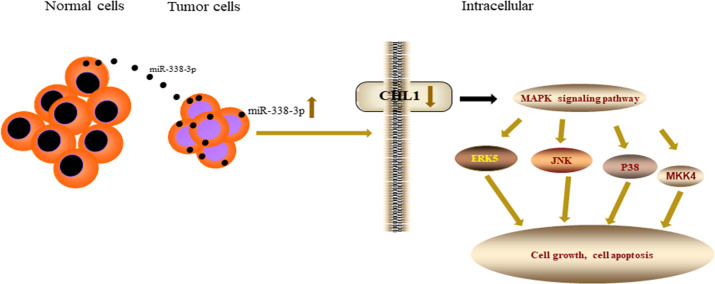
miR-338-3p was transferred by exosomes to NSCLC cells to inhibit CHL1 expression by inactivating MAPK signaling pathway to regulate the tumor cells metastasis.

In conclusion, we systematically investigated the oncogenic role of CHL1 in NSCLC development and metastasis. In addition, donor cells could transfer miR-338-3p to recipient cells. Furthermore, exosomal miR-338-3p suppresses tumor cells proliferation and induces apoptosis by inhibiting CHL1 through MAPK signaling inactivation. Exosomal miR338-3p/CHL1/MAPK signaling pathway axis provides a novel strategy for the diagnosis and therapy of NSCLC in the future (Fig. 8).

## Materials and methods

### Collection of raw data

Raw data and clinical characteristics of TCGA-NSCLC cohorts were downloaded by R package. Raw data contained the complete expression profile of CHL1 and miR-338-3p. The clinical characteristics included age, sex, TNM, clinical stage, survival time, and survival status. Raw data of GSE114711 and GSE11803 were collected from GEO (gene expression omnibus). GSE114711 contained 26 NSCLC plasma samples published in 2018. GSE111803 contained five patient samples and five healthy controls published in 2019.

Serums of patients with NSCLC were enrolled from Liaoning Cancer Hospital & Institute. All patients enrolled in this study signed informed consent documentation. The experiment was approved by the Institutional Review Board of China Medical University (no. 51 in 2017) and conducted using methodologies conforming to the standards set by the Declaration of Helsinki.

### High-throughput sequencing

A total of 14 serum samples were collected, including 7 NSCLC serum samples and 7 healthy serum samples. Samples were analyzed via Illumina platform (BioMarker, Beijing, China) and then constructed an miRNA database to obtain the raw data of exosome miRNAs. Adjusted *P* < 0.05 and |log_2_FC| > 1.5 (fold change) were set as the criterion for differently expressed exosomal miRNAs.

### Cells culture and transfection

Human normal lung epithelial cells (BEAS-2B) were cultured in RPMI-DMEM containing 20% FBS (fetal calf serum). Human NSCLC cell lines (A549 and SK-MES-1) were cultured in RPMI-1640 and RPIM-MEM medium containing 10% FBS, respectively. OE-CHL1 and OE-NC were purchased from Genechem (Shanghai, China). siRNA sequences against CHL1 were inverted into lentiviral vector GV248 (Shanghai Genechem Co., Ltd, China). miR-338-3p-mimics, NC, and miR-338-3p-inhibitor plasmids were designed from Syngeneiech (Beijing, China). The sequences were as follows:

**Table:** 

siRNA-CHL1:	5ʹ- TGCCGATATAACTCAAGTA -3'
siRNA-NC:	5ʹ-TTCTCCGAACGTGTCACGT-3'
miR-338-3p-mimics:	5ʹ-UCCAGCAUCAGUGAUUUUGUUG-3'
miR-NC:	5ʹ-UUGUACUACACAAAAGUACUG-3'
miR-inhibitor:	5ʹ-CAACAAAAUCACUGAUGCUGGA-3'

### Cells transfection

1 × 10^5^ to 1.5 × 10^5^ cells were seeded into six-well plates per well. After growth at 24 h, cells were washed twice by PBS. Culture medium containing siRNA, jetPRIME reagent (Ployplus transfection, USA), and jetPRIME buffer (Ployplus transfection, USA) was added to each well. The cells after growth 24 h later were collected for the following experiments.

### Western blotting

Western blotting was performed following procedures as described previously [43]. Polyclonal rabbit anti-human CHL1 (ABcam 106269, ABcam, China), anti-β actin (ABcam 8226, ABcam, China), anti-CD63 (ABcam 134045, ABcam, China), anti-TSG101 (ABcam 125011, ABcam, China), anti-ERK5 (ABcam 40809, ABcam, China), anti-JNK (ABcam 124956, ABcam, China), anti-MEK4 (ABcam 33912, ABcam, China) and anti-p38 (ABcam 170099, ABcam, China) at 4 °C overnight. After washing three times with 1× PBST, the membranes were incubated with a horseradish peroxidase-conjugated secondary antibody (ABcam 6721 and Abcam 6789, ABcam, China), ABcam, China for 1 h. The signal was detected with enhanced chemiluminescence (Thermo Scientific™ 34076, USA). ImageJ was used to analyze the integrated density values.

### Real-time PCR

Total RNA was collected by RNAiso Plus (TAKARA 9108). Reverse transfection was performed according to the instructions (TAKARA 638313). The reaction conditions consisted of buffer/samples and enzyme at 37 °C for 60 min and 85 °C for 5 min, followed by adding 90 μl ddH_2_O to bring the total volume to 100 μl. RT-PCR was conducted by the instructions of TAKARA RR820A kit. U6 was chosen as internal controls. The primers of miR-338-3p were designed and synthesized by Takara Biomedical Technology (Shenyang, China). The sequences of primers were as follows:

**Table:** 

pre-mir-338:	AACAATATCCTGGTGCTGAGTG
miR-338-3p:	TCCAGCATCAGTGATT
CHL1:	Forward: CCTCCTGTTAAAATTCTCAA
	Reverse: GGTTCTGGATTTCCTTTAG
U6:	Forward: TGGAACGCTTCACGAATTTGCG
	Reverse: GGAACGATACAGAGAAGATTAGC

Relative transcription levels of CHL1 and miR-338-3p were calculated based on 2^−∆∆Ct^ method with U6.

### Cell proliferation assay

A total of 100 μl cell suspension and 20 μl MTS solution (Promega G3580, China) were seeded in per well for 96-well plates. After 2 h, per well was tested at 490 nm. The proliferation rates of cells with different treatments were investigated at 0, 24, 48, and 72 h. The experiment was repeated three times.

### Cell apoptosis assay

The apoptotic rates of cells with different treatments were tested by Annexin V-APC/7-AAD (KGA1016, China) according to the protocols. The cells were harvested via pancreatin without EDTA. After washing twice by 1× PBS, cells were resuspended by 500 μl binding buffer, 5 μl Annexin V-APC and 5 μl 7-AAD. After culture in dark for 5−15 min, the apoptotic rates of cells were detected by FCM (Flow cytometry, Germany). The experiment was repeated three times.

### Dual luciferase assay

The wild and mutant type 3ʹ UTRs (untranslated regions) of CHL1 were synthesized and cloned into pmiR-RB-Report plasmid (RIBOBIO, Guangzhou, China). The plasmids were transfected into A549 cells and SK-MES-1 cells with miR‐338‐3p-mimics or NC (negative control). A total of 100 μl 1× PLB was added to per well to harvest the cell lysis buffer. The Dual Luciferase Assay (Promega E1910, China) was applied to analyze the luciferase activity in 48 h after transfection. The experiment was repeated three times independently.

### Exosome isolation and identification

FBS was ultracentrifuged at 12,000 × *g* for 16 h to delete exosomes. A549 cells and SK-MES-1 cells were cultured with mediums containing 10% exosome-deleted FBS. Conditioned media were collected and centrifuged at 500 × *g* for 10 min at 4 °C, followed by 15,000 × *g* for 30 min to discard cellular debris. The supernatants were ultracentrifuged at 120,000 for 1 h and then washed by PBS (phosphate buffer solution). The pellets were ultracentrifuged at 120,000 for 1 h at 4 °C and then resuspended in PBS. For transmission electron microscopy, exosomes were fixed with 5% glutaraldehyde and placed on carbon-coated copper grids. Then the grids were covered using 2% phosphotungstic acid solution for 2 min. Exosome size was detected by NanoFCM (Xiamen, China).

### GW4869

A549 cells and SK-MES-1 cells were respectively divided into two groups: GW4869 group and control group. Cells were seeded into culture dishes. Upon reaching 60−70% confluence, cells were washed twice by 1× PBS and cultured by medium containing 10 μM GW4869 for 48 h. Cells cultured by medium containing 0 μM GW4869 were taken as the control group. Cells with no treatment were taken as the blank group.

### Co-culture of exosomes and cells

After transfection with miR-338-3p-mimics and miR-338-3p mimics-NC, exosomes from BEAS-2B cells were collected. A549 cells and SK-MES-1 cells were seeded in a 24-well plate and upon reaching 60−70% confluence, exosomes from BEAS-2B cells were added to the medium for co-culture for 48 h. BEAS-2B cells co-cultured with A549 cells and SK-MES-1 cells in the transwell plates. BEAS-2B cells were seeded in the apical chamber of the transwell plates at 1−1.5 × 10^5^ per well and A549 cells and SK-MES-1 cells were seeded in the basolateral chamber of the transwell plates at 1−1.5 × 10^5^ per well. The volume of the complete medium in the apical chamber was 200 μl and the volume of the complete medium in the apical chamber was 500 μl. The following assays continued after co-culture 48 h.

### Statistical analysis

Statistical analyses were performed using SPSS 21.0 software. The unpaired *t* test was used for comparisons between different groups. Differences between two or more groups were analyzed by ANOVA (analysis of variance). *P* < 0.05 was considered to be statistically significant.

## Data Availability

The data that support the findings of this study are available on request from the corresponding author.
