# Correction: Exosomal miR-338-3p suppresses non-small-cell lung cancer cells metastasis by inhibiting CHL1 through the MAPK signaling pathway

**DOI:** 10.1038/s41419-022-04933-3

**Published:** 2022-05-19

**Authors:** Wen Tian, Xianglin Yang, He Yang, Meiwen Lv, Xinran Sun, Baosen Zhou

**Affiliations:** 1 Department of Clinical Epidemiology, First Affiliated Hospital, China Medical University, Shenyang, China; 2 Department of Epidemiology, School of Public Health, China Medical University, 110122 Shenyang, Liaoning China

**Keywords:** Non-small-cell lung cancer, Apoptosis

Correction to: *Cell Death and Disease* 10.1038/s41419-021-04314-2, published online 30 October 2021

The original version of this article contained a mistake in Table 2. The authors rechecked the data and improved the clinical stage carefully. They found the duplication of N classification and lymphatic metastasis and the error of the clinical stage. Due to the mistake of statistics and no clinical significance of N classification and clinical stage, they decided to delete the data of N classification of Table 2 (line 22–24) and amend the data of clinical stage (line 28–31), which modifications have no effects on the results of this study. The authors apologize for the error. The amended table can be found below. The original article has been corrected.

**Table 2:** The clinical characteristics of exosomal miR-338-3p in NSCLC patients.

Characteristics	exosomal miR-338-3p		*P* value
	High expression	Low expression	
Age			0.155
<60	16	15	
≥60	12	23	
Gender			0.833
Male	14	18	
Female	14	20	
Pathological classification			
Lung adenocarcinoma	24	14	**<0.001**
Lung squamous carcinoma	1	15	
Others	3	9	
Smoking			0.541
Yes	9	15	
No	19	23	
Drinking			
Yes	5	10	0.418
No	23	28	
T			0.566
T1+T2	16	19	
T3+T4	12	19	
M			**0.018**
M0	17	12	
M1	11	26	
Clinical stage			0.379
I+II	11	11	
III+IV	17	27	
Lymphatic metastasis			**0.030**
Yes	16	31	
No	12	7	

Bold values indicate statistical significance.

